# Supplementary material for: The interactome of intact mitochondria by cross-linking mass spectrometry provides evidence for coexisting respiratory supercomplexes
Source: Mol Cell Proteomics. 2017 Dec 8;17(2):216–32. doi: 10.1074/mcp.RA117.000470 (PMC5795388; doi:10.1074/mcp.RA117.000470)
Supplement: Supplemental Data [file supp_RA117.000470_133922_0_supp_23978_fzffwf.zip › spectra_annotation/mito_spectra_annotation/390-1-13-1-48-1.pdf]

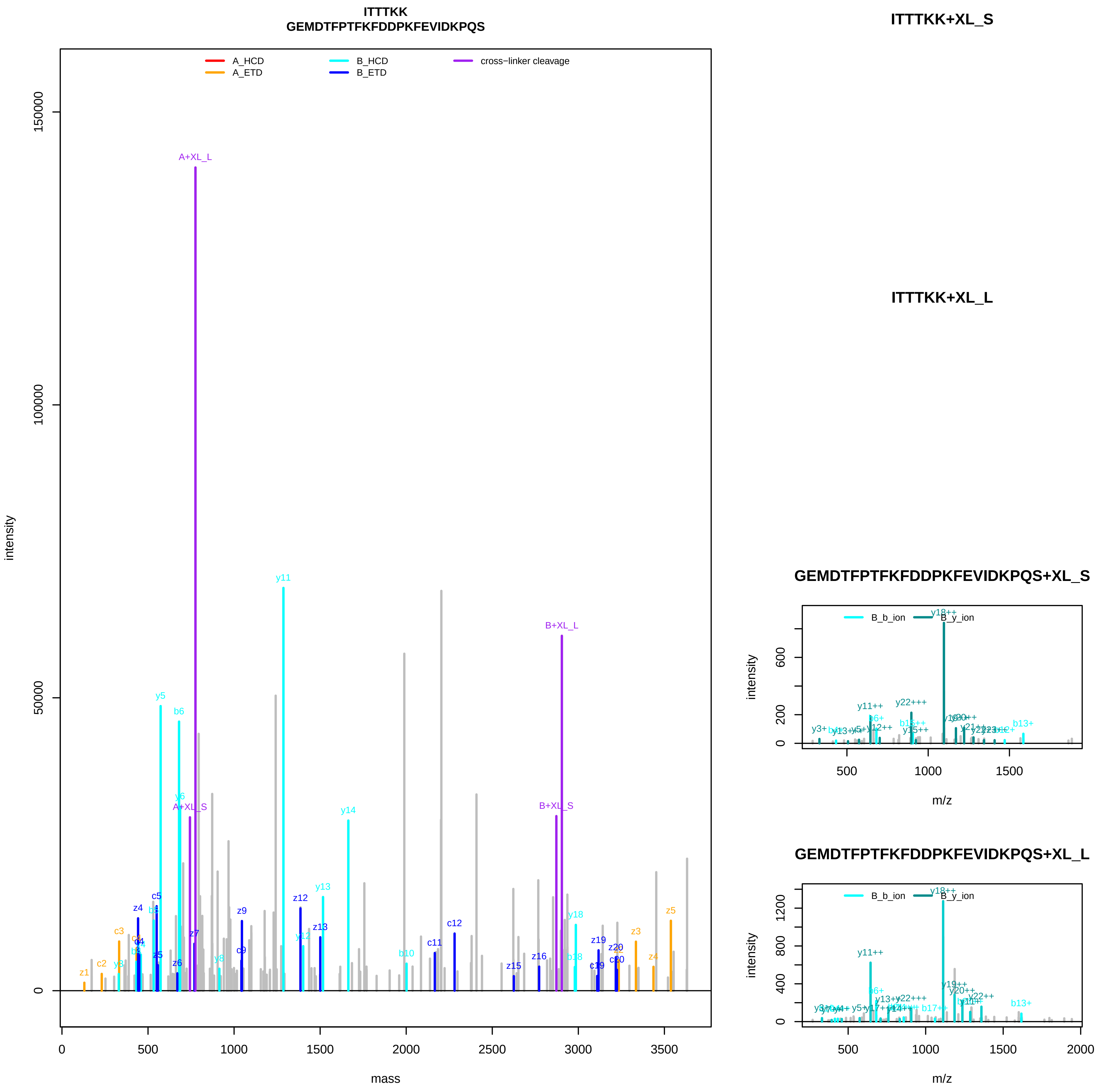

ITTTKK
GEMDTFPTFKFDDPKFEVIDKPQS
A_HCD
A_ETD
B_HCD
B_ETD
cross−linker cleavage
intensity
mass
A+XL_L
A+XL_S
B+XL_L
B+XL_S
y11
y5
b6
y14
y13
z12
z13
c12
c11
z9
c5
z4
z16
z15
z19
z20
c20
y18
z3
z5
z4
c3
c2
z1
ITTTKK+XL_S
ITTTKK+XL_L
GEMDTFPTFKFDDPKFEVIDKPQS+XL_S
GEMDTFPTFKFDDPKFEVIDKPQS+XL_L
B_b_ion
B_y_ion
y18++
y11++
y22+++
b13+
m/z
